# Silica-Nanocoated Membranes with Enhanced Stability and Antifouling Performance for Oil-Water Emulsion Separation

**DOI:** 10.3390/membranes15020041

**Published:** 2025-02-01

**Authors:** Mengfan Zhu, Chengqian Huang, Yu Mao

**Affiliations:** Department of Biosystems and Agricultural Engineering, Oklahoma State University, Stillwater, OK 74078, USA; mengfan.zhu@okstate.edu (M.Z.); chengqian.huang@okstate.edu (C.H.)

**Keywords:** emulsion separation, fouling, chemical vapor deposition, ultrasmooth, silica

## Abstract

Despite the potential of glass fiber (GF) membranes for oil-water emulsion separations, efficient surface modification methods to enhance fouling resistance while preserving membrane performance and stability remain lacking. We report a silica nanocoating method to modify GF membranes through a vapor deposition method. The high smoothness (<1 nm r.m.s.) and high conformality of the vapor-deposited silica nanocoatings enabled the preservation of membrane microstructure and permeability, which, combined with the enhanced surface hydrophilicity, led to an oil rejection rate exceeding 99% and more than a 40% improvement in permeate flux in oil-water emulsion separations. Furthermore, the silica nanocoatings provided the membranes with excellent wet strength and stability against organic solvents, strong acids, oxidants, boiling, and sonication. The silica-nanocoated membrane demonstrated enhanced fouling resistance, achieving flux recovery higher than 75% during repeated oil-water emulsion separations and bovine serum albumin and humic acid fouling tests.

## 1. Introduction

Large amounts of oil-contaminated wastewater are produced by various industries, including petroleum, food, and textiles [1,2]. To prevent adverse impacts on ecosystems and human health, oil must be removed before the discharge of wastewater into the environment [3,4,5]. However, the emulsification of the oil phase in the wastewater makes it difficult to be removed using traditional oil/water separation methods [6]. For example, the flotation process becomes highly time-consuming due to the dispersion of oil droplets smaller than 10 μm [7]. Currently, membrane filtration is considered a promising method for the rapid and effective removal of emulsified oil. In addition, membrane separation offers advantages such as high oil removal rates, low energy consumption, a compact footprint, and easy automation [7,8,9,10].

Both inorganic and polymer membranes have been investigated for separating oil–water emulsions [11]. While polymer membranes are generally advantageous due to mechanical flexibility and cost-effectiveness [12,13,14], they are less resistant to harsh conditions such as high temperature, extreme pH, and concentrated organic compounds [15]. For example, a crosslinked polybenzimidazole membrane lost permeability completely after exposure to the organic solvent N,N-dimethylformamide due to structural distortion [16]. In such cases, glass fiber (GF) membranes—an inorganic membrane that combines the advantages of polymeric membranes with enhanced chemical and thermal stability [15,17]—become a promising candidate.

The primary obstacle limiting the widespread application of membrane technology in oil-water separation is membrane fouling [7]. To improve resistance to oil fouling, GF membranes have been modified with hierarchical nano- and microstructures to attain underwater superoleophobicity. However, studies have suggested that such structured rough surfaces are prone to fouling by common organic contaminants such as proteins [18] and humic acid (HA) [19], because the high surface area and ridge-valley structures trap foulants through physical interlocking [20].

While smooth-surface oleophobic GF membranes have been reported [17,21,22,23], few have demonstrated resistance to fouling by oil, HA, and proteins. In addition, most of them relied on surface modification with polymeric coatings [17,21,22,23], which compromised the GF membrane’s inorganic characteristics and, consequently, its stability. For example, a copolymer of poly(dimethylsiloxane) and poly(N,N-dimethylaminoethyl methacrylate)-coated GF membrane showed unstable water flux when the pH of the feed varied [21]. Furthermore, these surface modification processes often involved toxic solvents such as piranha solution [17] and complex synthetic routes, which could limit their scalability.

Inorganic silica nanocoatings are promising candidates due to their abundant surface hydroxyl groups, which offer strong hydrophilicity and underwater oleophobicity [24,25]. Silica coatings are commonly synthesized through the hydrolysis of tetraethyl orthosilicate (TEOS), but this process typically yields nanoparticle structures with high surface roughness [24,25,26,27]. Non-uniform particle distribution and incomplete coverage can negatively impact the antifouling performance [28].

We report synthesis of ultrasmooth silica nanocoatings using chemical vapor deposition (iCVD) and their application in fabricating antifouling GF membranes for oil-water emulsion separation. As a solventless process, iCVD eliminates liquid surface tension, which can cause uneven and incomplete coating coverage, and allows precise control of coating thickness at the nanoscale, thus forming uniform, conformal, and ultrathin coatings for membrane modification [29,30,31]. We studied the effects of CVD silica nanocoating on the morphology, wettability, and permeability of GF membranes. The oil-in-water emulsion separation performance was also investigated. To evaluate the membrane’s antifouling properties, we measured the flux variation during filtration of the diesel-in-water emulsion, HA solution, and bovine serum albumin (BSA) solution. The fouling ratio and flux recovery between the pristine and nanocoated membranes were compared. The nanocoated membranes’ stability against ultrasonication, organic solvents, oxidants, extreme pH, and hydrothermal conditions was examined.

## 2. Materials and Methods

### 2.1. Materials

GF membranes with a nominal pore size of 0.7 μm (grade F) were supplied from Whatman (Cleves, OH, USA). BSA (66 kDa) and tert-butyl peroxide (TBP, 98%) were purchased from Sigma-Aldrich (St. Louis, MO, USA). Humic acid (HA) and dodecyl sulfate sodium (SDS, >99%) were purchased from Acros Organics (Waltham, MA, USA). 3-(Trimethoxysilyl)propyl methacrylate (TMSPMA, >98%) was purchased from TCI America (Portland, OR, USA). Hydrogen peroxide (H_2_O_2_, 30%), sodium hydroxide (NaOH, >97%), and phosphate-buffered saline (PBS, 10X) were purchased from Fisher Scientific (Waltham, MA, USA). Sulfuric acid (H_2_SO_4_, 96%) and acetone (99.5%) were supplied by Pharmco Aaper. Diesel was purchased from a local gas station (Stillwater, OK, USA).

### 2.2. Preparation of Silica-Nanocoated GF Membranes

The silica nanocoatings were synthesized using iCVD [29] of poly(TMSPMA) (PTMSPMA), followed by annealing. During iCVD, the initiator TBP was vaporized at room temperature and fed into a reactor through a mass flow controller (MKS Instruments, Andover, MA, USA; model 1479A). The monomer of TMSPMA was vaporized at 80 °C and fed into the reactor through a needle valve (Swagelok, Solon, OH, USA). The flow rates of TBP and TMSPMA were maintained constantly at 0.85 sccm and 0.17 sccm, respectively. Inside the reactor, an array of Ni80/Cr20 filaments was resistively heated to 230 °C, while the stage substrate was maintained at 35 °C by circulating water. All temperatures were measured using directly-attached thermal couples (Omega Engineering, Norwalk, CT, USA; type K). The pressure inside the reactor was controlled at 0.09 torr using a butterfly valve (MKS Instruments, Andover, MA, USA; model 253B). The deposition process was monitored by measuring the increase in coating thickness on a flat silicon wafer placed beside the membrane samples. The thickness of PTMSPMA coatings synthesized on the membranes was estimated to be 100 nm and 300 nm, based on the coating thickness on the silicon wafer. After iCVD, the PTMSPMA-coated GF membranes were annealed in air at 400 °C for 1 h using a muffle furnace (Thermo Scientific, Waltham, MA, USA; type FB1315M). The annealed membranes of 100 nm and 300 nm PTMSPMA are designated as GF/Si100 and GF/Si300, respectively.

### 2.3. Membrane Characterizations

Membrane morphology was observed using an FEI Quanta 600 field-emission scanning electron microscope (SEM). The chemical composition of the coatings was analyzed using Fourier transform infrared (FTIR) spectra, collected over a range of 400–4000 cm^–1^ at 4 cm^–1^ resolution using a Nicolet 6700 spectrometer. The water contact angle (CA) of the coatings on silicon wafers was measured using the standard sessile drop method with a 4 μL water droplet, using a goniometer system (Ramé-hart, Cedar Knolls, NJ, USA; model 250F1). Since the membrane hydrophilicity cannot be measured by water CA due to the spontaneous penetration of water droplets into the GF membranes, a qualitative method reported in the literature [17,24,32] was used for evaluation. This method speculates that when a water droplet makes contact with the hydrophilic membrane surface, it divides into two parts: one spreading along the top surface and the other penetrating into the membrane’s bulk. The membrane with more water penetration into its bulk is considered more hydrophilic. In this method, 10 µL of water dyed with alizarin red was placed on the membrane surface using a micropipette. The stained (wetting) area on the membrane’s top and reverse sides was visually observed. The underwater oil CA (UWOCA) was measured by floating a drop of diesel under the membranes.

### 2.4. Emulsion Separation Experiments

A dead-end filtration setup was used for all microfiltration experiments, including the water permeability test, emulsion separation test, and antifouling test. The membrane was wetted with pure water before testing. The transmembrane pressure was kept constant at 2 in Hg using a vacuum system.

For the emulsion separation test, oil-in-water emulsion was prepared by mixing diesel and water in the 1:99 volume ratio with the addition of 50 mg L^–1^ SDS. The emulsion was stirred for 2 h before use. The flux J (L m^–2^ h^–1^) was calculated using the following equation:

(1) $$J=\frac{V}{A\times t}$$

where *V* (L) is the filtrate volume through an effective filtration area *A* (m^2^) over a filtration time *t* (h). The oil rejection rate *R* (%) was calculated using the following equation:

(2) $$R=\left(1-\frac{C_{1}}{C_{0}}\right)\times 100\%$$

where *C*_0_ (ppm) and *C*_1_ (ppm) are the diesel concentrations in the feed and the filtrate, respectively. The diesel concentration was measured using gas chromatography-mass spectrometry (Shimadzu, Kyoto, Japan; GCMS-QP2010). The diesel droplets were visually observed under the optical microscopy (Leica Microsystems, Wetzlar, Germany; DMI3000 M).

### 2.5. Membrane Stability Tests

The membrane’s stability against chemical, physical, and hydrothermal treatments was measured following methods in the literature [17,33]. Chemical treatments were applied by immersing the coated membranes in H_2_SO_4_ (96%), NaOH (0.1 M), H_2_O_2_ (30%), and acetone (99.5%) at room temperature for 24 h, followed by a thorough cleaning with pure water. Physical treatments were applied by immersing the membranes in a water bath of ultrasonic cleaner (Branson Ultrasonics, Brookfield, CT, USA; 2510) at room temperature for 1 h. Hydrothermal treatments were applied by immersing the membranes in boiling water for up to 9 h. After these treatments, BSA static adsorption onto membranes was conducted to assess the integrity of the silica coatings across the membrane surface. For BSA static adsorption, a membrane with a diameter of 21 mm was immersed in 2 mL BSA solution (0.1 g L^–1^ in PBS, pH = 7.4) at room temperature for 12 h. The BSA concentration in the resulting solution was then determined using a Micro BCA Protein Assay Kit (Thermal Scientific, Waltham, MA, USA).

### 2.6. Membrane Antifouling Tests

Membrane anti-oil-fouling tests were performed by filtrating the oil-in-water emulsions (1% *v/v* diesel, 50 mg/L SDS). During filtration, the filtrate collected was weighed every 5 min, and the membrane was washed every 35 min by rinsing it in deionized water.

For the BSA and HA fouling tests, 1 g L^–1^ BSA in pH7.4 PBS solution [34] and 0.01 g L^–1^ HA in pH7.0 aqueous solution [35] were prepared before use. First, filtration started with pure water until a stable flux, J_0_ (L m^–2^ h^–1^), was reached. Then, filtration proceeded with the BSA or HA solution, and the normalized flux, J/J_0_, was reported. The fouled membrane was cleaned through a backwash procedure using 0.5 L of pure water at a transmembrane pressure of 5 in Hg.

## 3. Results and Discussion

### 3.1. Membrane Preparation and Characterization

**Figure 1:**
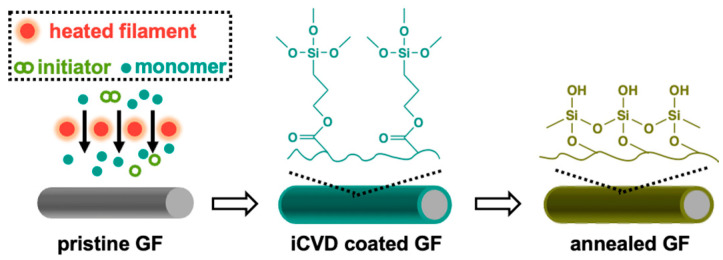
Preparation of silica-nanocoated GF membranes. The PTMSPMA was coated on GF membranes using iCVD, followed by in-air annealing at 400 °C for 1 h.

**Figure 2:**
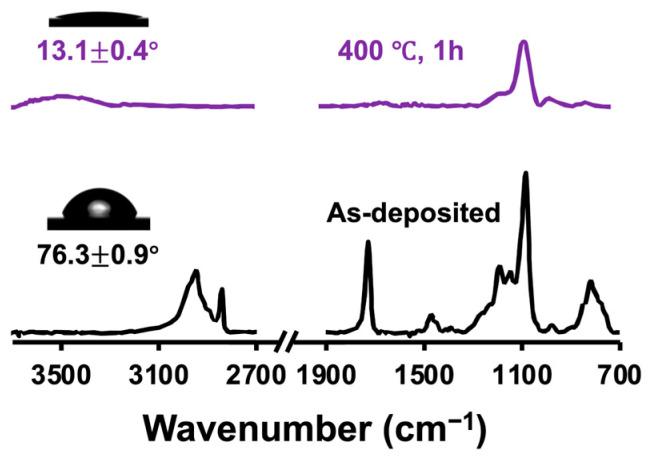
FTIR spectra and water contact angle of as-deposited PTMSPMA and the silica nanocoatings formed after annealing.

Ultrasmooth silica-nanocoated GF membranes were prepared using iCVD of PTMSPMA followed by annealing (Figure 1). During iCVD, the monomer TMSPMA was fed into the reactor along with the initiator TBP. The initiator was thermally decomposed into tert-butoxy radicals [36] to initiate the polymerization of TMSPMA, forming PTMSPMA nanocoatings around each fiber of the GF membranes. The chemical composition of the as-deposited PTMSPMA was confirmed using FTIR spectroscopy (Figure 2). The absorbance peaks at 2945, 2840, and 1724 cm^−1^ are attributed to the stretching vibrations of -CH_2_, -CH_3_, and C=O groups, respectively [37,38]. The peaks at 820 and 1080 cm^−1^ are assigned to the symmetric and asymmetric bending vibrations of Si-O-C [39,40], while the split peak ranging from 1130 to 1220 cm^−1^ is assigned to the bending vibration of Si-C [39]. After annealing at temperatures higher than 400 °C, the absorbance peaks at 2945, 2840, and 1724 cm^−1^ vanished, and the peak at 1080 cm^−1^ transferred to a broad doublet at 1050 cm^−1^ attributed to the vibrations of Si-O-Si [41], confirming the removal of the organic moieties and formation of Si-O-Si crosslinked structures under high-temperature annealing. Meanwhile, a huge hump appeared around 3300–3700 cm^−1^, corresponding to the hydroxyl group (-OH) [42].

**Figure 3:**
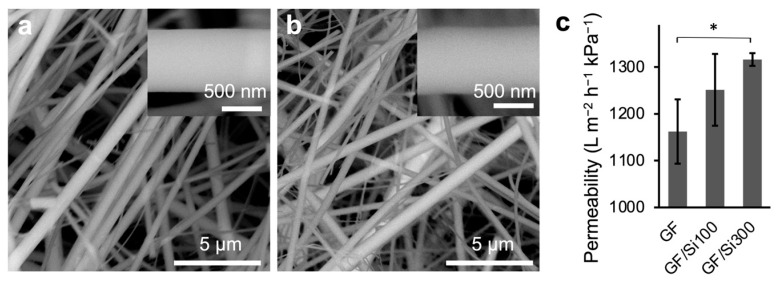
SEM images of (**a**) pristine GF and (**b**) GF/Si300. The insets show an enlarged view of the fibers. (**c**) Pure water permeability of the pristine, GF/Si100, and GF/Si300 membranes. Differences were considered statistically significant when * *p* < 0.05.

The membrane morphology before and after silica nanocoating was examined using SEM. The pristine GF membrane consisted of overlapped fibers (Figure 3a), forming slit-shaped pores, which offer an advantageous balance between permeability and selectivity [43,44]. The slit-shaped pore geometry was preserved after surface modification (Figure 3b), which was attributed to the high conformality of the iCVD-deposited PTMSPMA to the fiber contours [30,31]. In addition, the fiber surface maintained its smoothness after the silica nanocoating was applied, indicating a minimal impact of the nanocoating process on surface texture. This is evident in the magnified view, which shows a consistently smooth fiber surface.

**Figure 4:**
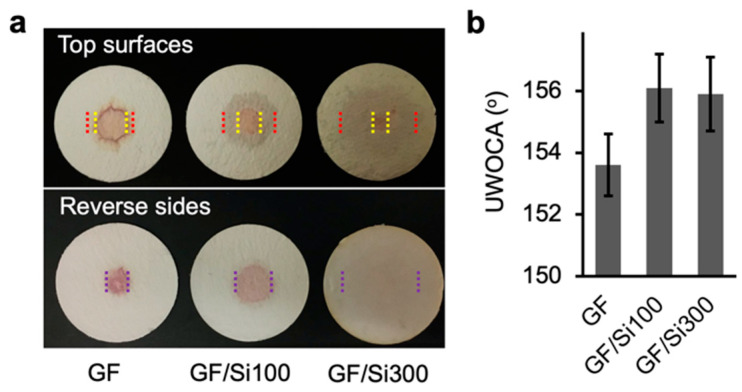
(**a**) Photos of membranes with 10 μL dyed water. The yellow, red, and purple lines indicate the stained (wetting) area of the top surface, intermediate layer, and bottom surface. (**b**) The underwater oil contact angle (UWOCA) of membranes.

The preservation of membrane pore openings after silica nanocoating ensured no reduction in membrane permeability. As shown in Figure 3c, the pure water permeability was 1162 ± 69 L m^–2^ h^–1^ kPa^–1^ for the pristine membrane, and it increased to 1251.2 ± 77 and 1316 ± 69 L m^–2^ h^–1^ kPa^–1^ for the GF/Si100 and GF/Si300 membranes, respectively. The increased permeability was attributed to the increased surface hydrophilicity, which was qualitatively verified by observing the spreading behavior of a dyed solution on the membrane [17,24,32]. Compared with the pristine membrane, the silica-nanocoated membranes showed a larger wetting area at the top surface (Figure 4a, red lines) and on the reverse side (purple lines), indicating higher hydrophilicity. The improved hydrophilicity was attributed to the increased abundance of hydroxyl groups on the silica nanocoating compared with the borosilicate of the pristine GF membrane.

The membranes’ underwater oleophobicity was examined using UWOCA measurement. The pristine GF membrane, characterized by its inherently hydrophilic porous structure, exhibited excellent underwater oleophobicity, as demonstrated by a high UWOCA of 153° (Figure 4b). The silica nanocoating further improved the underwater oleophobicity, increasing the UWOCA to 156°. This improvement was attributed to enhanced surface hydrophilicity, which facilitated the formation of a hydration layer on the membrane surface, effectively resisting the spread of oil droplets.

### 3.2. Emulsion Separation Performance

**Figure 5:**
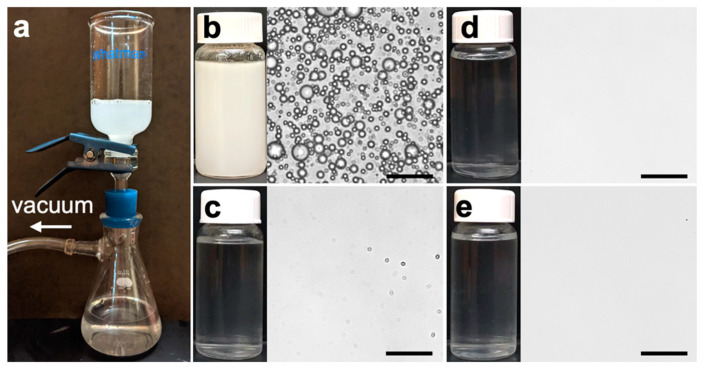
(**a**) Photos of the separation apparatus. Microscopy images of (**b**) SDS-stabilized diesel-in-water emulsion and the filtrates of (**c**) pristine GF, (**d**) GF/Si100, and (**e**) GF/Si300 membranes. Scale bar = 30 μm.

**Figure 6:**
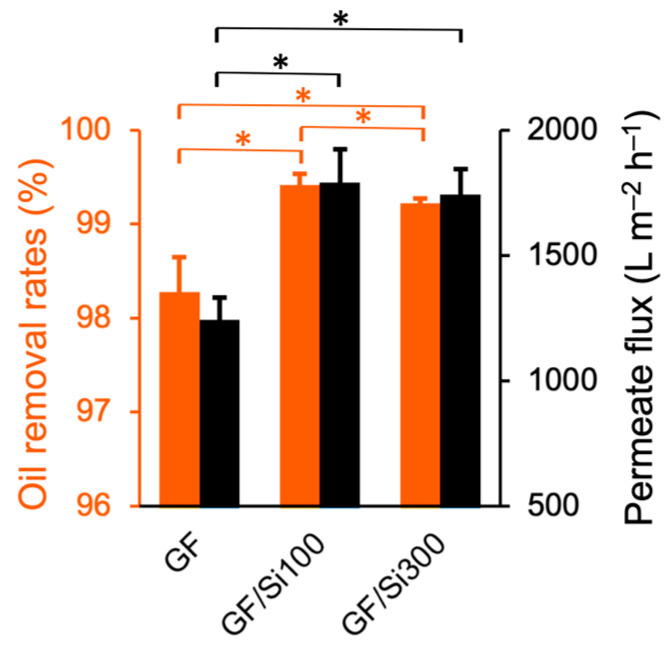
Oil removal rates and permeate flux of pristine and silica-nanocoated membranes in emulsion separation. Differences were considered statistically significant when * *p* < 0.05.

The membrane separation performance was measured by filtering diesel-in-water emulsions using a dead-end apparatus under a transmembrane pressure of 2 inHg (Figure 5a). The emulsion contained oil droplets ranging from hundreds of nanometers to tens of microns in diameter (Figure 5b). The filtrates from the silica-nanocoated GF membranes were free of visible oil droplets (Figure 5d,e), in contrast to the filtrate from the pristine membrane, which contained a noticeable amount of oil droplets (Figure 5c). By measuring the diesel concentration in the filtrate, the oil rejection rate of the pristine membrane was determined to be 98.28%, which improved to 99.42% and 99.22% for the GF/Si100 and GF/Si300 membranes, respectively (Figure 6). In addition to enhanced selectivity, the silica-nanocoated membranes also demonstrated a substantial 40.3–44.1% increase in filtrate flux compared with the pristine membrane. The improved separation performance was attributed to enhancements in both membrane permeability and underwater oleophobicity.

### 3.3. Membrane Stability

**Figure 7:**
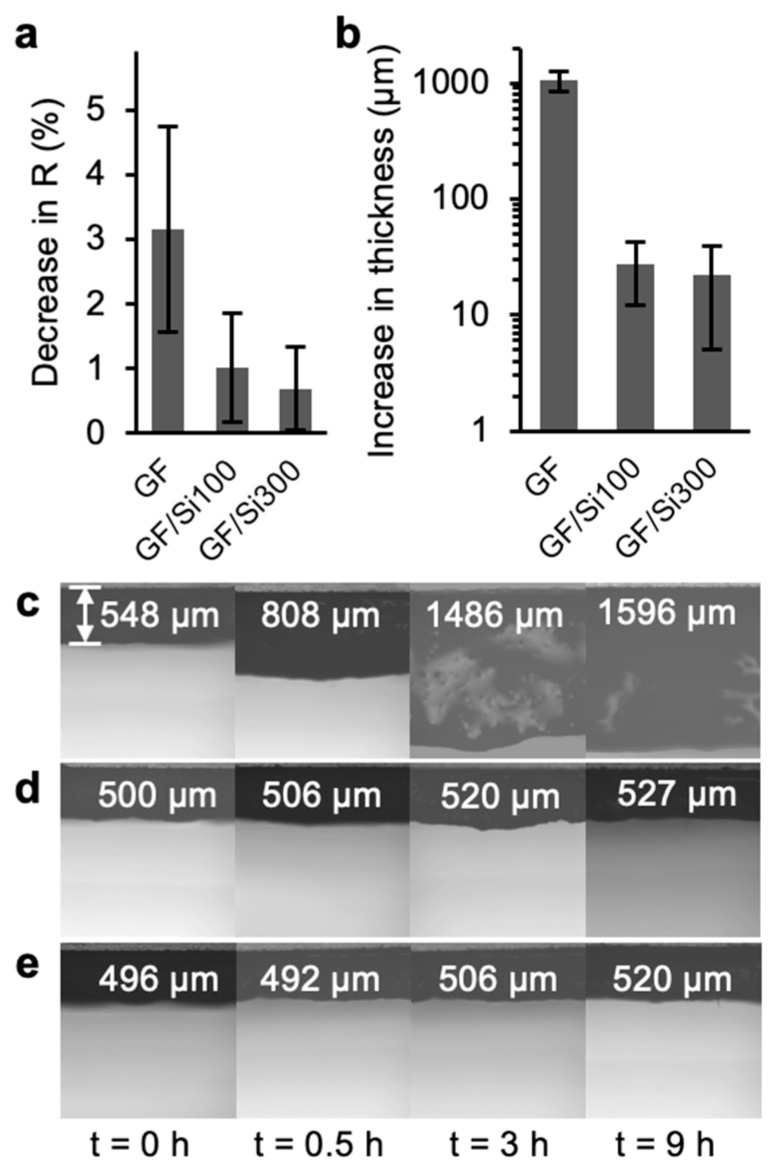
(**a**) Decrease in oil rejection rates and (**b**) increase in membrane thickness of pristine and nanocoated membranes after the water boiling test. Optical microscopy images of the cross-section of the (**c**) pristine GF, (**d**) GF/Si100, and (**e**) GF/Si300 membranes after boiling for different periods.

We investigated the membrane’s hydrothermal stability by measuring oil rejection rates after treatment with boiling water. The pristine membrane exhibited a 3.15% decrease in oil rejection rates after treatment, whereas the GF/Si100 and GF/Si300 membranes showed significantly smaller reductions of 1.01% and 0.69%, respectively (Figure 7a). The thickness of the pristine membrane increased significantly after the boiling treatment; in contrast, the nanocoated membranes maintained compact fiber packing with only minimal thickness increase (Figure 7b,c). Most likely, the water boiling induced fiber displacement and pore enlargement in the pristine membrane, which resulted in the decrease of oil rejection. Notably, the silica nanocoatings served as both a binder for the glass fibers and a surface modifier, leading to a significant improvement in the membrane’s wet strength.

**Figure 8:**
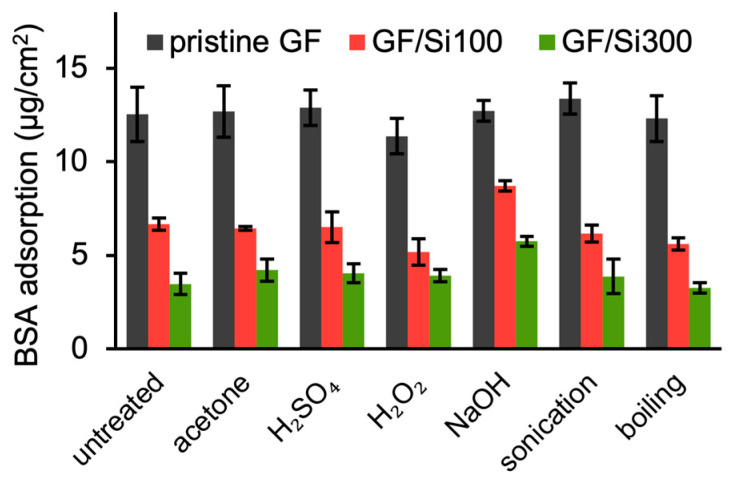
BSA adsorption on membranes after harsh treatments, including 24-h immersion in acetone (99.5%), H_2_SO_4_ (96%), H_2_O_2_ (30%), and NaOH (0.1 M), 1-h sonication in water, and 12-h immersion in boiling water.

BSA adsorption tests of the membranes were conducted after exposure to harsh conditions. As shown in Figure 8, the untreated GF/Si100 and GF/Si300 membranes showed BSA adsorption of 6.66 ± 0.34 and 3.47 ± 0.56 µg cm^–2^, respectively, significantly lower than the adsorption on the pristine membrane. After 24 h soaking in acetone, the silica-nanocoated membranes maintained low BSA adsorption, demonstrating stability against organic solvents. The nanocoated membranes also withstood treatments with acid and oxidants that are commonly used for membrane cleaning [45]. While the traditional organic binder of GF membranes had limited chemical resistance [17,21], the silica nanocoating provided the GF membranes with excellent chemical resistance, along with a significant improvement in wet strength. In addition, the BSA fouling on silica-nanocoated membranes remained unchanged after sonication, which simulated filtration shear force, and immersion in boiling water, which simulated high-temperature wastewater conditions.

### 3.4. Fouling Resistance to Oil, BAS, and HA

**Figure 9:**
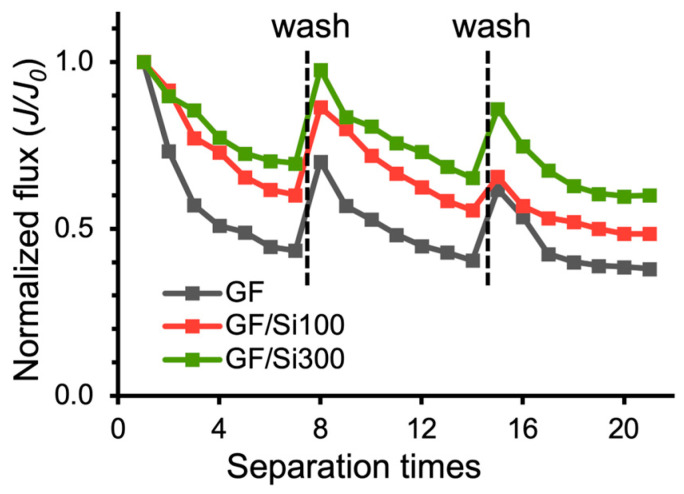
The anti-oil-fouling performance of pristine and silica-nanocoated GF membranes, as measured by normalized flux during the repeated filtrations of oil-in-water emulsions (SDS: 50 mg/L, diesel: 1% *v*/*v*).

The silica nanocoatings improved the membrane’s anti-oil-fouling performance. As shown in Figure 9, the permeate flux of the pristine GF membrane rapidly decreased in the second filtration cycle of the oil-in-water emulsion, and the flux ratio dropped to 0.44 at cycle seven. The GF/Si100 and GF/Si300 membranes exhibited a much slower flux decline in the repeated filtrations, and the J/J_0_ ratio was at 0.60 and 0.70, respectively, indicating higher resistance to the adhesion of oil. In addition, the nanocoated membranes achieved significantly higher flux after the backwash, indicating the nanocoating’s ability to facilitate the removal of the attached oil foulants. The improved resistance to oil fouling was attributed to the silica nanocoating’s superhydrophilicity, which promoted the formation of a surface hydration layer that acted as a physical barrier to prevent oil adhesion [46].

**Figure 10:**
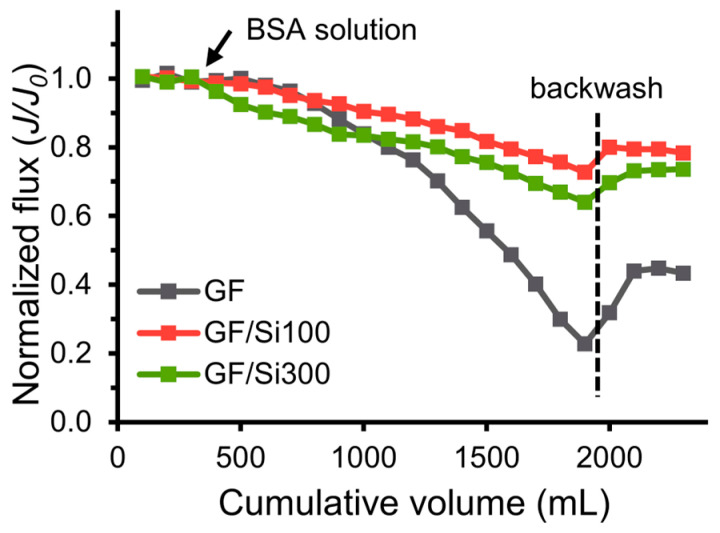
Normalized flux of the pristine and nanocoated membranes versus the cumulative permeate volume in the BSA fouling test.

The silica-nanocoated GF membranes exhibited higher resistance to BSA fouling than the pristine membrane (Figure 10). Though both the pristine and nanocoated membranes showed similar flux decline during the initial filtration, the flux decline of nanocoated membranes was much slower than that of the pristine membrane at the later stages (after cumulative permeate volume of 1000 mL). The flux decline was attributed to the BSA adsorption on fiber surface, which decreased the inter-fiber space and increased the hydraulic resistance of the membrane [43]. When the normalized flux of the pristine membrane dropped below 0.2, the J/J_0_ ratio of the GF/Si100 membranes was 0.73, more than three times higher, demonstrating the nanocoated surface’s significantly stronger resistance to BSA adsorption. In addition, the J/J_0_ ratio of the GF/Si100 membrane recovered to 79.3% after the membrane backwash, nearly twice that of the pristine membrane (41.31%). These results indicated the nanocoated membranes’ potential for enhanced reusability and a prolonged lifespan against fouling.

**Figure 11:**
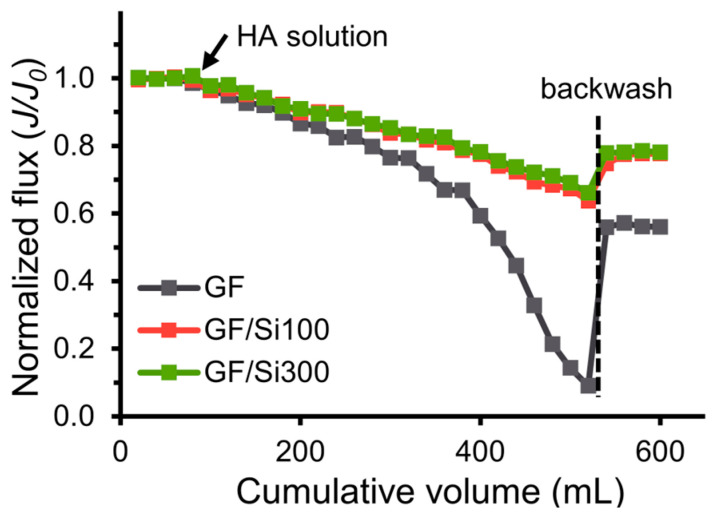
Normalized flux of the pristine and nanocoated membranes versus the cumulative permeate volume in the HA fouling test.

Figure 11 shows the membranes’ flux variation during the fouling test of HA. The normalized flux of GF/Si100 and GF/Si300 membranes dropped to 0.64 and 0.66, respectively, whereas that of the pristine membrane dropped significantly to 0.09. This result demonstrated that the silica nanocoating improved surface resistance to HA adsorption. Since the membrane surface architectures did not change after coating, this improvement was attributed to the enhanced surface hydrophilicity of silica nanocoatings and the electrostatic repulsion between the silica’s silanol groups and the HA’s carboxyl groups, which were both negatively charged at neutral pH [47,48].

As shown in Table 1, compared with silica-decorated membranes and other modified glass fiber membranes, the silica-nanocoated membranes exhibit superior permeate flux, comparable oil removal rates, fouling resistance, and chemical and thermal stability. The combination of these properties makes the nanocoated membranes promising for the practical filtration of oil-water emulsions.

## 4. Conclusions

Silica-nanocoated membranes were successfully developed through iCVD deposition and annealing of nanocoatings on GF membranes. The silica nanocoatings featured conformal coverage over the fibers, preservation of membrane microstructure, tunable thickness, and enhanced surface hydrophilicity. The silica-nanocoated membranes demonstrated improved permeate flux and enhanced oil rejection compared to the pristine membrane in oil-water emulsion separations. Additionally, the silica nanocoatings significantly improved the membrane’s wet strength while maintaining its chemical resistance. The silica-nanocoated membrane achieved flux recovery greater than 75% during repeated oil-water emulsion separations and BSA and HA fouling tests. The combination of separation performance, chemical and thermal stability, and fouling resistance demonstrates the potential of silica-nanocoated GF membranes for efficient and durable use in oil-water emulsion separations.

## Figures and Tables

**Table 1:** Performance comparison of silica-decorated and modified glass fiber membranes.

Membrane	Flux (L m^−2^ h^−1^ bar^−1^)	Oil Removal Rate	Fouling	Stability	Ref
Nano-mica-coated PVDF membrane	~720	99.5%	-	-	[49]
Silica-nanoparticle-modified polysulfone membrane	400–1000	-	flux recovery 55–75% after BSA		[27]
Polyethyleneimine-modified glass fiber membrane	900–1000	99.7%	-	sustained chemical solvent and boiling treatment	[17]
Silica & PDMS-coated glass fiber membrane	~10,000	-	-	-	[21]
Silica-nanocoated glass fiber membrane	26,445–25,747	99.2–99.4%	flux recovery 79.3% after BSA	sustained chemical solvent and boiling treatment	This work

^“^-^”^ indicates that data are not available.

## Data Availability

The original contributions presented in this study are included in the article. Further inquiries can be directed to the corresponding author.
